# Analysis of the alcohol drinking behavior and influencing factors among emerging adults and young adults: a cross-sectional study in Wuhan, China

**DOI:** 10.1186/s12889-019-6831-0

**Published:** 2019-04-30

**Authors:** Wanrong Lu, Jingdong Xu, Anne Winifred Taylor, Bridgette Maree Bewick, Zhen Fu, Nanjin Wu, Ling Qian, Ping Yin

**Affiliations:** 1 Department of Epidemiology and Biostatistics, School of Public Health, Tongji Medical College, Huazhong University of Science and Technology, 13 Hangkong Road, Wuhan, 430030 People’s Republic of China; 2 Hubei Institute for Health Education, Zhuodao Quan Road No.6, Hongshan District, Wuhan, 430079 China; 3 Population Research & Outcome Studies, Discipline of Medicine, The University of Adelaide, SAHMRI, North Terrace, Adelaide, 5006 Australia; 4 Division of Psychological and Social Medicine, Leeds Institute of Health Sciences, School of Medicine, University of Leeds, Leeds, LS2 9NL UK; 5 Chinese Center for Health Education, PR Ministry of Health, Building 12, Block 1, Anhua Xili, Anwai Dajie, Chaoyang District, Beijing, 100011 China

**Keywords:** Alcohol, Drinking behavior, Influencing factors, Adolescent, Transition, Emerging adulthood, Wuhan, Emerging adults

## Abstract

**Background:**

The relationship between alcohol use in adolescents and young adults and outcomes has not been widely researched in China. The aim of the current study was to understand the current status of drinking behavior of Chinese youth transitioning into adulthood.

**Methods:**

The cross-sectional study included 1634 participants between 18 and 34 years of age. The participants were randomly chosen from 13 administrative districts in Wuhan, and invited to complete a questionnaire. Univariate analysis was performed to describe the demographic distribution of alcohol consumption and the association with drinking status. Stepwise Logistic regression analysis was undertaken analyzing the factors influencing the drinking behaviors. The data were weighted to the population in Wuhan and analyzed using SAS version 9.3.

**Results:**

For our sample of emerging and young Chinese adults the prevalence of drinking alcohol was 45.84%. The non-drinkers predominated, accounting for 54.16% and light drinkers accounted for 42.94%, while moderate and heavy drinkers were in the minority (2.90%). The earlier the age of first alcohol drinking or the age of first being intoxicated, the greater the likelihood of being a moderate or heavy drinker. People with high emerging adulthood were more likely to have moderate or heavy drinking behaviors. The logistic regression analysis indicated that heavy drinkers were more likely to not be married and to be classified as high emerging adulthood.

**Conclusions:**

Our findings suggested that the drinking pattern should be further evaluated over time to explore the ways in which social and cultural factors shape the drinking route of this age group. Effective drinking behavior prevention and interventions and appropriate guidance should be formulated to establish an appropriate attitude towards drinking alcohol and develop a drinking behavior which is conducive to physical and mental health between this particular demographic.

## Background

According to the World Health Organisation’s 2014 estimate, 5.1% of the global disease and injury burden can be attributed to drinking alcohol [1]. In recent years, the analysis of international trends and investigations into the determinants of substance use has revealed alcohol consumption as one of the substances typically used and abused by adolescents and young adults [2–4]. Heavy drinking has largely been a phenomenon of emerging and young adulthood [5, 6], which places individuals at risk for adverse health and social effects [7–9]. It is believed this vulnerability is in part due to the dramatic life changes in relationships, employment, accommodation and roles of the individual as they transition into adulthood [10, 11]. Harmful drinking behaviors in young years have been associated with greater risk of severe psychiatric and other drinking problems in adults [12]. Reducing high risk drinking patterns remain a major public health challenge [13].

In China, drinking alcohol is a widely accepted cultural tradition, especially during rituals, festivals, social gatherings, commercial occasions and special activities [14]. Based on the estimates of the Chinese National Bureau of Statistics, alcohol production and consumption in China has seen a sharp growth in recent decades [15, 16]. The acceleration in growth is the highest internationally [17]. There is an inseparable relationship between alcohol consumption growth and the Chinese cultural context [18, 19].

Compared with other countries, early research on alcohol in China focused on the etiology of adults, mainly containing the physiological and clinical analysis. In recent years, investigations have begun to pay closer attention to alcohol-related health harm (e.g. mortality, chronic disease, cancer) [17, 20–22] and social problems (e.g. drunk driving, drunk crime, injuries) [23–25]. A local and national literature examining drinking behaviors and drinking motivations associated with alcohol consumption in China is emerging [26–28]. These studies have chiefly focused on adults, even the studies investigating younger drinkers have concentrated on adolescent or student populations [29, 30]. There remains a relative paucity of studies on drinking prevalence, drinking behaviors and its related factors in China [31]. It is extremely rare for alcohol studies to concentrate attention on emerging and young adults groups in China. Accordingly, our research is of significance as the first attempt to explore the current status of drinking behaviors of emerging and young adults under the specific social and cultural background in China.

Within the literature no one age is identified as the exact point of transition into adulthood. Within the literature there is broad agreement that the point of transition occurs somewhere between late adolescence and the age of more than 30 [32–34]. Given this background the current study defined emerging and young adulthood as being between 18 to 34 years old. The outcomes of the current study will inform the development of prevention and early-intervention programs for emerging and young adults in China.

## Methods

The data were derived from a trans-national population-based survey covering four cities (Moscow, Ilorin, Wuhan, Montevideo) in four countries. In the current paper, the Wuhan data was used to investigate the drinking behavior among emerging adults and young adults (18–34 years old) under a specific social and cultural background in China. The method part refers to the published papers by Anne and Bridgette [35].

### Ethics approval and consent to participate

This study received ethical review and approval from the Hubei Provincial CDC (Hubei Provincial Society for Health Promotion and Cigarette-smoking Control, HBPHP & CCS-2014-01). Before further investigation, the interviewers conducted household confirmation, informed consent, and investigation management issues. The interviewers must introduce the content and purpose of the survey according to specific introductions and obtain verbal informed consent from all participants. Participants could terminate the interview at any time, or choose not to answer any question. Confidentiality of information was assured. Approval for the research was obtained by community leaders, appropriate government officials and other important related personnel. Households with alcohol and substance abuse could be referred to recognized hospitals/caregivers for proper management.

### Data source and participants

Wuhan is the capital of Hubei Province, and at the time of the survey it had a population of approximately 9.8 million and a geographical area of about 8467 km^2^. Wuhan was pragmatically selected as a research site based on its diversity. Wuhan is divided into 13 administrative regions, and the multi-stage random sampling was performed across each region. The sample size of each administrative district was proportional to the population size. Communities were randomly selected in each administrative district, followed by random household selection. In each randomly selected household, the person with the most recent birthday, aged between 18 and 34 years, who lived in Wuhan for at least six months, was eligible and was invited to participate in the study. Prior to the main survey, 33 pilot interviews were conducted. The pilot assessed participants’ understanding of the questions and length of the interview.

Data collection lasted for 186 days (2014.10.25–2015.04.29). Door-knocking interviews were conducted in Chinese and the Wuhan local dialect by the interviewers who were local college students with specialized training. The data was obtained through face-to-face interviews, which is better for mutual understanding between interviewee and interviewer. Interviewers read out the questions and recorded via paper-and-pencil. Interviews were conducted in an environment where respondents felt most comfortable and where their privacy was respected. The average interview time was 15 min. In total 1675 individuals were approached and 1675 participants agreed to be interviewed. After screening 1646 were identified as eligible to be interviewed. Of these 1642 interviews were completed. Two interviews were excluded due to insufficient completion of demographic information; demographic information is necessary for weighting. A further six cases were excluded due to incomplete drinking information. A total of 1634 questionnaires were included in the current analysis. The response rate was therefore 99.3%.

### Drinking questionnaire

Main alcohol questions included: (1) Have you ever consumed alcohol (excluding sips). (2) How old were you the first time you had a drink of an alcoholic beverage (excluding sips)? (3) How old were you the first time you got drunk? (4) During the past 12 months, how often did you drink beer, wine, spirits (e.g. vodka, gin, whisky, brandy), huangjiu or any other alcoholic beverage, even in small amounts? (5) During the past 12 months, how many alcoholic drinks did you have on a typical day when you drank alcohol? (6) In the past 12 months, how often have you ever gotten drunk? (Drunk refers to walking unsteadily, blurred vision, slurred speech, nausea, vomiting or any other symptoms)? (7) Drinking alcohol may impose various effects on people. When you are drinking, on what extent of the following statement may fit your situation (Always, usually, sometimes, seldom, never)? The effects including positive and negative effects, such as feel happy, feel relaxed, become aggressive toward other people, and so on.

Overall quantity-frequency (i.e. usual frequency of drinking by usual number of drinks consumed per drinking occasion) was calculated by multiplying the responses to the above two questions (how much and how many) with 25 or more drinks (coded as 25), 19–24 drinks (coded as 21.5), 16–18 drinks (coded as 17), 12–15 drinks (coded as 13.5), 9–11 drinks (coded as 10), 7–8 drinks (coded as 7.5), 5–6 drinks (coded as 5.5), 3–4 drinks (coded as 3.5), 2 drinks (coded as 2), 1 drink (coded as 1) and less than 1 full drink (coded as 0.5). The annual number of drinks was calculated by multiplying the responses to the question on how many days did you drink alcohol with the response from how many drinks did you have. The variables were recorded into four drinking status groups: 0 drinks = Abstainers; > 0 but less than 365 drinks/year = Light drinkers; 365–729 drinks/year = Moderate drinkers; 730 or more drinks/year = Heavy drinkers. No distinction was made between lifetime abstainers and former drinkers with alcohol consumption assessed during the past 12 months only. These four groups were collapsed into two groups of non-drinkers and current drinkers. ‘Non-drinkers’ referred to those who have never drunk before (‘Lifetime abstainers’) and those who have not drunk in the past 12 months (‘Former drinkers’). ‘Current drinkers’ referred to those who have either drunk more than half a cup of alcohol at least once in the past 12 months or those who have drunk any quantity of alcohol at least twice in the past 12 months.

Pure alcohol intake (grams per day) was calculated by multiplying the responses to questions about how often and how much—and then the units were converted (the calculation was made according to the standard glass of specific alcoholic beverages, 1 ml alcohol = 0.789 g). Respondents were provided with visual references to beverage specific drink sizes in order to facilitate reporting of number of drinks in standard sizes.

The interview also included an assessment of emerging adulthood which included the following three statements: a) I have reached adulthood. b) I am financially independent of my parents. c) I am emotionally independent of my parents. Questions were answered on a five-point Likert scale: 5 = strongly agree; 4 = agree; 3 = neither agree nor disagree; 2 = disagree; 1 = strongly disagree. Responses were totaled with 3–11 points indicating low emerging adulthood, 12–13 points indicating medium emerging adulthood, and 14–15 points indicating high emerging adulthood. The emerging adulthood statements were based on the work of Arnett and Padilla-Walker [36] and adapted to fit common assumptions within China. Scores were reversed so that lower emerging adulthood scores indicated lower emerging adulthood.

### Statistical analysis

In this study, the data were ranked weighted as per the stratified target population information provided by Hubei Provincial Institute for Health Education in 2014 by Administrative District, age, gender and selection probability. The weight value distribution range was 0.285–4.300. The aim was to minimize bias and ensure that samples were representative; the weighted demographics of the sample were consistent with the local demographics.

The data base was built with EpiData version 3.1, and the logical verification and statistical analysis of the data were carried out using the 9.3 version of the SAS statistical analysis package. Demographic characteristics included age, sex, marital status, education level, current student status and employment status. Group comparison of categorical data was using CMH-***χ***^***2***^ test. The rank test was used for group comparison of grade data. Simple univariate analysis was performed to describe the demographic distribution of alcohol consumption and the association analysis of drinking status. Stepwise Logistic regression analysis was undertaken analyzing the factors influencing the drinking status among the population between 18 and 34 years of age in Wuhan, controlling for the socio-demographic characteristics and adult tendency. In addition, the relationship between emerging adulthood and drinking behavior was assessed. The significance was set at *p* < 0.05.

## Results

### The demographic and emerging adulthood distribution of alcohol consumption

**Fig. 1:**
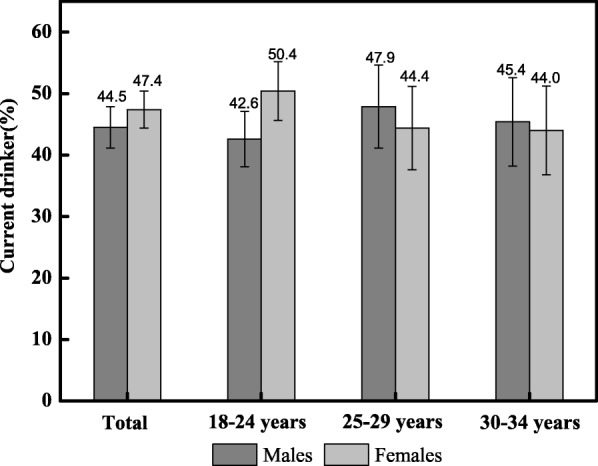
The proportion of current alcohol drinkers by age group

A total of 1634 people were included in the current analysis. Of these 749 (45.8%) were current drinkers. Chi-square statistics were calculated to assess the drinking distribution difference between different demographic characteristics and emerging adulthood (Table 1). Significant differences were observed in the drinking distribution between different marital status, current student status and emerging adulthood. The proportion of married people drinking was lower than those not married (*χ*^*2*^ = 10.14, *p* = 0.0015); Students had a higher proportion of drinking than non-students (*χ*^*2*^ = 5.09, *p* = 0.0240); People with high emerging adulthood had a higher proportion of drinking (*χ*^*2*^ = 10.09, *p* = 0.0064). Figure 1 displays the current drinking proportion for the three age groups. Although not significant, younger females (18–24 years) were more likely to drink than males.

**Table 1:** The demographic and emerging adulthood distribution of alcohol consumption

Variables	Non-drinking		Drinking		*χ* ^*2*^	*p*
	n	% (95% CI)	n	% (95% CI)		
Marital status					10.14	*0.0015*
Married	424	58.6 (54.9–62.1)	300	41.4 (37.9–45.1)		
Not married	461	50.7 (47.4–53.9)	449	49.3 (46.1–52.6)		
Current student status					5.09	*0.0240*
Yes	161	48.6 (43.3–54.0)	170	51.4 (46.0–56.7)		
No	724	55.6 (52.9–58.2)	579	44.4 (41.8–47.1)		
Emerging adulthood					10.09	*0.0064*
Low	219	58.4 (53.4–63.3)	156	41.6 (36.7–46.6)		
Medium	218	49.1 (44.5–53.7)	226	50.9 (46.3–55.5)		
High	343	48.9 (45.2–52.6)	359	51.1 (47.4–54.8)		

Legend: 95% CI was calculated by Newcombe-wilson method

### Drinking behavior

Participants were divided into four categories: non-drinkers, light drinkers, moderate drinkers and heavy drinkers. The non-drinkers predominated, accounting for 54.2% of the population, followed by light drinkers (42.9%), moderate drinkers (1.7%) and heavy drinkers (1.2%). The drinking status between different demographic characteristics and emerging adulthood are shown in Table 2. It revealed a statistically significant difference between drinking status and marital status, current student status, as well as emerging adulthood. Non-students had a higher proportion of moderate or heavy drinkers (***χ***^***2***^ = 4.24, *p* = 0.0395); People who were not married had a higher proportion of heavy drinkers (***χ***^***2***^ = 7.22, *p* = 0.0072); People with high emerging adulthood had a higher proportion of heavy drinkers (*χ*^*2*^ = 7.36, *p* = 0.0067).

**Table 2:** Difference analysis of drinking status

Variables	Non- drinkers		Light drinkers		Moderate drinkers		Heavy drinkers		*χ* ^*2*^	*p*
	n	% (95% CI)	n	% (95% CI)	n	% (95% CI)	n	% (95% CI)		
Marital status									7.22	*0.0072*
Married	424	58.6 (54.9–62.1)	279	38.5 (35.1–42.1)	13	1.8 (1.1–3.0)	8	1.1 (0.6–2.2)		
Not married	461	50.7 (47.4–53.9)	423	46.5 (43.3–49.7)	15	1.6 (1.0–2.7)	11	1.2 (0.7–2.2)		
Current student status									4.24	*0.0395*
Yes	161	48.6 (43.3–54.0)	162	48.9 (43.6–54.3)	1	0.3 (0.1–1.7)	7	2.1 (1.0–4.3)		
No	724	55.6 (52.9–58.2)	540	41.4 (38.8–44.1)	27	2.1 (1.4–3.0)	12	0.9 (0.5–1.6)		
Emerging adulthood									7.36	*0.0067*
Low	219	58.4 (53.4–63.3)	148	39.5 (34.7–44.5)	6	1.6 (0.7–3.4)	2	0.5 (0.1–1.9)		
Medium	218	49.1 (44.5–53.7)	210	47.3 (42.7–51.9)	11	2.5 (1.4–4.4)	5	1.1 (0.5–2.6)		
High	343	48.9 (45.2–52.6)	340	48.4 (44.8–52.1)	7	1.0 (0.5–2.0)	12	1.7 (1.0–3.0)		

Legend: 95% CI was calculated by Newcombe-wilson method

The results of the rank test (Table 3) indicated that males with an early first drinking age (z = 2.49, *p* = 0.0129) or a first drunk age (z = 2.46, *p* = 0.0138) before 15 years of age, had a higher proportion of moderate or heavy drinkers than that after the age of 15; No significant relationship was observed for females (small cell sizes). Overall, the earlier the first drinking age, the higher the level of current drinking (z = 2.57, *p* = 0.0101). When exploring the relationship between drunkenness and negative effects among current drinkers, the incidence of intoxication was 33.6% for those who linked drinking with negative effects, while for people who did not consider drinking with negative effects, the incidence of intoxication was 18.7%, a difference of 15.0% (95% CI (0.081–0.205), *p* < 0.0001).

**Table 3:** The relationship between the first drinking/drunk age and current drinking status

	Age at the first drinking		*z*	*p*	Age at the first drunk		*z*	*p*
	≤14	≥15			≤14	≥15		
Male			2.49	*0.0129*			2.46	*0.0138*
Light drinkers	15 (78.9)	299 (94.0)			4 (57.1)	143 (88.8)		
Moderate drinkers	3 (15.8)	11 (3.5)			2 (28.6)	12 (7.5)		
Heavy drinkers	1 (5.3)	8 (2.5)			1 (14.3)	6 (3.7)		
Total	19	318			7	161		
Female			−0.68	0.4972			−0.60	0.5470
Light drinkers	7 (100.0)	313 (93.7)			3 (100.0)	117 (88.6)		
Moderate drinkers	0 (0.0)	12 (3.6)			0 (0.0)	8 (6.1)		
Heavy drinkers	0 (0.0)	9 (2.7)			0 (0.0)	7 (5.3)		
Total	7	334			4	132		
Overall			2.57	*0.0101*			1.77	0.0765
Light drinkers	21 (80.8)	612 (93.9)			7 (70.0)	260 (88.7)		
Moderate drinkers	4 (15.4)	23 (3.5)			2 (20.0)	20 (6.8)		
Heavy drinkers	1 (3.9)	17 (2.6)			1 (10.0)	13 (4.5)		
Total	26	652			10	293		

### Drinking behaviors and emerging adulthood

Stepwise regression methodology was used to determine the model for demographic characteristics and drinking status; age, marital status and emerging adulthood were positively related to drinking status (Table 4). There was a significant difference in marital status (*p* < 0.0001) and higher emerging adulthood (*p* = 0.0030), with heavy drinkers more likely to be people not married, and with higher emerging adulthood.

**Table 4:** Adjusted odds ratios (OR) for drinking status

Variables	*β*	*SE*	*OR*	*95% CI*	*p*
Intercept1	−4.3410	0.2320			*< 0.0001*
Intercept2	−3.4131	0.1504			*< 0.0001*
Intercept3	−0.0695	0.0605			0.2508
Age					
18–24			1.000		
25–29	0.1492	0.0831	1.545	(1.137–2.101)	0.0724
30–34	0.1368	0.0966	1.526	(1.076–2.166)	0.1568
Marital status					
Married			1.000		
Not married	0.3016	0.0758	1.828	(1.358–2.461)	*< 0.0001*
Emerging adulthood					
Low			1.000		
Medium	0.2004	0.0676	1.039	(0.779–1.385)	0.1192
High	0.1192	0.0765	1.325	(1.021–1.721)	*0.0030*

Legend: Adjusted by age, marital status and emerging adulthood

**Fig. 2:**
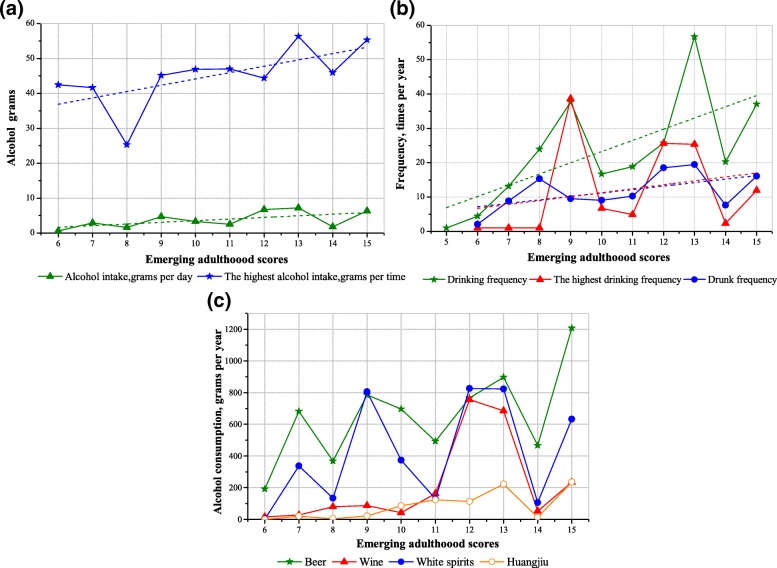
The relationship between alcohol grams (**a**), drinking/drunk frequency (**b**), and four alcoholic beverages’ consumption (**c**) with emerging adulthood scores (For current drinkers)

Figure 2, (a) demonstrates the daily average alcohol intake and the highest alcohol intake significantly increased as emerging adulthood scores increased. The result of rank correlation analysis showed the correlation coefficient of emerging adulthood and the alcohol intake (grams per day) was 0.56 (*p* = 0.0897). The correlation coefficient of emerging adulthood and the highest alcohol intake (grams per time) was 0.76 (*p* = 0.0111). Similarly, in the Fig. 2 (b), drinking frequency, the highest drinking frequency and the frequency of being drunk significantly decreased as adult tendency scores decreased. The correlation coefficient of emerging adulthood and the drinking frequency was 0.70 (*p* = 0.0165). The correlation coefficient of emerging adulthood and the highest drinking frequency was 0.52 (*p* = 0.1276). The correlation coefficient of emerging adulthood and frequency of being drunk was 0.52 (*p* = 0.1221). Figure 2, (c) showing that the alcohol consumption of beer was significantly higher than other types of alcoholic beverages, followed by white spirits, wine and huangjiu. The annual alcohol consumption of four types of alcoholic beverages (beer, wine, white spirits and huangjiu) showed a upward trend with the increasing emerging adulthood scores.

## Discussion

This is the first study aimed at evaluating the drinking behaviors and its related factors among emerging and young adults in China. The current study suggests that the majority of people aged 18–34 years living in Wuhan who consume alcohol could be considered ‘light drinkers’. Although the incidence of heavy drinking is not high among this population when extrapolated to the number of individuals across China, this represents a substantial challenge for public health. In addition, we cannot predict whether light or moderate drinkers will engage in dangerous drinking behaviors in the future. Public health prevention and intervention efforts in China should strengthen and develop an appropriate attitude towards drinking alcohol. There is also a need to develop effective interventions for the relatively few, but substantial in number, heavier drinkers in order to minimize the costs associated with problematic drinking behavior.

The influence of gender on drinking behavior has been demonstrated in many studies. Some alcohol studies reported that males were more likely to consume alcohol than females, and females were more likely to be abstainers [37–39]. In our gender analysis, females had a slightly higher current drinking proportion than males (2.82% higher). This finding may be related to recent changes in economic and social status resulting in females developing a stronger independent consciousness, increasing levels of employment, and increasing social activities and communication opportunities. Other studies have highlighted that male excesses are no longer apparent along with the appearance of “narrowing of the gender gap” in particular time periods, geographical settings, age groups and drinking patterns [40–43]. Consistent with the findings that males had higher drinking frequency and exceeded females in rates of heavy drinking behavior in other studies, we found males had a higher proportion of moderate or heavy drinkers [17]. In addition, the results revealed students were more likely to report being consumers of alcohol, this is consistent with surveys from other countries [44, 45].

Previous studies have noted a first drinking age of 15 years or earlier was at elevated risk for alcohol abuse and dependence [46, 47]. The earlier the first drinking age, the probability of subsequently developing heavy drinking patterns and resultant health damage was markedly increased [48, 49]. Our results also indicated that, whether male or female, a first drinking age before 15 had a greater potential risk of engaging in heavy drinking when compared to people who started later than 15. Those who started drinking before 15 years of age were also more likely to be moderate or heavy drinkers. Publish research confirms that age of reporting first being drunk is related to the negative effects of drinking [50]. Early-onset drunkenness is associated with an increased risk of developing alcohol use disorder and signaled excess mortality risk [51]. Consistent with these earlier reports, our results showed that the younger the age of first drunkenness, the greater the likelihood of becoming a moderate or heavy drinker. Those who had ever been intoxicated were more likely to associate drinking with negative effects. Premature drinkers or premature drunks have been found to be more impulsive and adventurous, more extreme and poorly controlled, and report the occurrence of health problems and other risks in later life [47, 52, 53].

The current results concerning emerging adulthood found an association between emerging adulthood and drinking behaviors. Heavy drinkers were more likely to be people with higher emerging adulthood. Furthermore, people with higher emerging adulthood usually had more extreme drinking alcohol behavior with higher frequency. Previous studies suggested that young adults reduce risk drinking behavior to more moderate levels of alcohol use when adult roles and responsibilities are taken on [54–56]. Our results suggested an increase of alcohol use in those with higher emerging adulthood. This apparent contradiction between our study and the published literature may be the result of differences in culture between China and other countries. As emerging adults mature, they encounter more responsibility and play new roles. For current Chinese drinkers it can be difficult to avoid consuming alcohol, especially during social activities where there is a felt pressure to drink [57]. Greater social pressure and more drinking occasions can be related to trends in drinking motives, drinking opportunities and drinking behaviors.

Our research also indicated a link between the alcohol consumption of the four types of alcoholic beverages (beer, wine, white spirits and huangjiu) and emerging adulthood scores, which have shown that among the four most common types of alcoholic beverages in China, the annual alcohol consumption of beer was significantly highest, which supported the fact that China is one of the largest beer consuming countries in the world [58].

Study limitations include the fact that the study was cross-sectional, which restricts our assessments of the temporal relationships of the associations. The data was limited to one city and the results were not nationally representative. Concerns around the length of the structured interview and lack of availability of a validated brief assessment of ‘emerging adulthood’ meant the current study created a measure based on the work of Arnett and Padilla-Walker [36]. The study would have been strengthened had a standardized, validated, brief-assessment of emerging adulthood been available. Our definition of a heavy drinker precludes those who drink very occasionally but who on these occasions drink very heavily. Future research should aim to understand this important group of individuals. Study strengths include focusing on emerging adults and young adults aged between 18 to 34 years old, a large and targeted sample is conducive to formulating effective prevention and intervention. Probability-based random sampling methodology (stratified, clustered, systematic) was adopted to ensure the random selection of population. The data were weighted, ensuring findings were representative of the broader urban population. In addition, this self-report method can incorporate all alcohol consumption levels under the circumstance that many official statistics often do not record the alcohol consumption.

## Conclusions

The research focused on the alcohol drinking behaviors of this particular age group, and the findings of the present study strongly supported the significant impact of social and cultural background and age-specific characteristics on the drinking behaviors. For China,, China-specific influences together with assessment of adulthood level are important considerations when developing effective drinking interventions for emerging and young adults. Internationally, this country-specific study increases the understanding of Chinese drinking characteristics and provides valuable reference for international alcohol-related surveys.

## Abbreviations

HBPHP & CCS: Hubei Provincial Society for Health Promotion and Cigarette-smoking Control
